# High-Sensitivity Goos-Hänchen Shift Sensing via Surface Plasmon Resonance and Beam Displacement Amplification

**DOI:** 10.3390/s25051329

**Published:** 2025-02-21

**Authors:** Qian Li, Enze Xu, Xiaoliang Zhang, Jianguo Tian, Zhibo Liu

**Affiliations:** 1 The Key Laboratory of Weak Light Nonlinear Photonics, Ministry of Education, School of Physics and Teda Applied Physics Institute, Nankai University, Tianjin 300071, China; 2120230287@mail.nankai.edu.cn (Q.L.); stevenxez@gmail.com (E.X.); jjtian@nankai.edu.cn (J.T.); 2 State Key Laboratory of Photovoltaic Materials and Cells, Nankai University, Tianjin 300071, China; 3 Institute of Biomedical Precision Testing and Instrumentation, College of Artificial Intelligence, Taiyuan University of Technology, Jinzhong 030600, China; zhangxiaoliang@tyut.edu.cn; 4 The Collaborative Innovation Center of Extreme Optics, Shanxi University, Taiyuan 030006, China

**Keywords:** Goos–Hänchen shift, surface plasmon resonance, sensing, beam displacement amplification

## Abstract

Surface plasmon resonance (SPR) sensing technology has been widely utilized in fields such as biomedicine, food safety, and drug screening for real-time, rapid, and label-free detection of biomolecular interactions. However, conventional SPR sensing methods find it difficult to provide the necessary sensitivity and stability when detection applications go toward ultra-low concentrations and tiny molecular weight analytes. Here, we present a high-sensitivity Goos–Hänchen shift sensing based on SPR and beam displacement amplification technology (BDAT). The incorporation of BDAT significantly amplifies the magnitude of GH shift with remarkable stability, enhancing the sensing sensitivity by an order of magnitude. The sensor achieves a sensitivity of 3.62 × 10^4^ μm/RIU and a minimum detection limit of 3.10 × 10^−5^ RIU. Furthermore, both theoretical and experimental results demonstrate that GH shift sensing offers superior performance compared with traditional intensity-based SPR, particularly for low-concentration solutions. The BDAT approach amplifies GH shifts by at least 12 times, significantly improving sensitivity. With the use of SPR and BDAT, we are able to generate a large GH shift, which makes it easier to detect low concentrations and offers a wide range of possible uses in clinical diagnostics and biomedicine.

## 1. Introduction

Surface plasmon resonance (SPR) sensing technology is an optical sensing method highly sensitive to the refractive index of surface media, based on the resonance phenomenon between incident light and surface plasmons on a metal surface [1,2,3,4,5,6,7,8,9,10,11,12]. Its ability to detect biomolecular interactions in real-time, rapidly, and without labeling has garnered significant attention. Currently, SPR sensing exhibits broad application prospects in fields such as biomedicine, environmental monitoring, food safety, and drug screening [13,14,15]. However, as detection applications shift toward ultra-low concentrations and small molecular weight analytes, traditional SPR sensing techniques fail to meet the required sensitivity and stability [16].

**Figure 1:**
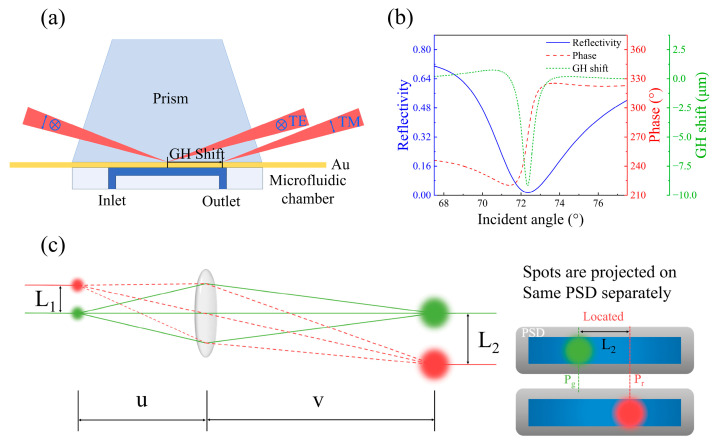
(**a**) Schematic diagram of the GH shift sensing. (**b**) Simulated results of reflectivity, phase, and GH shift signal variations. (**c**) Schematic of BDAT. Two spots separated by L_1_ are collected and magnified by a convex lens, resulting in an amplified spot separation of L_2_. The magnified spots are projected onto the position-sensitive detector (PSD) to determine their positions (Pg and Pr, respectively).

To address this challenge, researchers have been investigating novel approaches to enhance the sensitivity of SPR sensing. Among these, Goos–Hänchen (GH) shift sensing (Figure 1a) has emerged as a promising method, offering significant sensitivity improvements compared to traditional SPR sensing [17,18,19,20,21,22,23,24,25]. As shown in Figure 1b, unlike the gradual changes in intensity [26,27,28] or wavelength [29,30,31] of SPR sensing, the optical phase undergoes rapid variations when the reflectivity approaches zero, allowing phase-detection methods to improve sensitivity by several orders of magnitude [32,33]. Moreover, higher-order phase signals, such as GH shift, can further enhance sensitivity. The singular behavior of the phase in Fourier space under zero reflectivity leads to abrupt phase jumps, resulting in significant GH shift, making it an excellent choice for detecting ultra-low concentration biomolecules [19,20,21,22,23].

Subsequently, researchers achieved significant improvements in sensor performance by introducing two-dimensional materials, optimizing the thickness of the metal layer, and adjusting the optical configuration. Theoretically, Chen et al. [34] calculated the GH shift in a graphene-Ag composite structure, demonstrating a maximum sensor sensitivity of 6.3372 × 10⁶ μm/RIU. Han et al. [35] investigated the impact of heterostructures composed of blue phosphorene (BlueP)/transition metal dichalogenides (TMDCs) and graphene on GH shift. They achieved a maximum GH shift of −2361 λ with a sensitivity of 2.767 × 10⁷ λ/RIU, where λ = 632.8 nm. Nurzad Zakirov et al. [36] optimized a composite structure consisting of copper, TMDCs, and graphene. The structure with five layers of Wse_2_ and two layers of graphene exhibited a maximum sensitivity of 2.26 × 10⁶ μm/RIU at an excitation wavelength of 1024 nm. Experimentally, Wang et al. [25] proposed a 2D Ge_2_Sb_2_Te-Au composite structure. The GH shift sensor based on this structure achieved a sensitivity of 5.95 × 10⁴ μm/RIU and successfully detected crucial biomarker TNF-α at concentrations as low as 1 fM. Manel Hedhly et al. [37] designed a biosensor based on a symmetric metal cladding plasmonic waveguide structure, achieving a sensitivity of 2.33 × 10⁴ μm/RIU with a detection limit of 10⁻⁸ RIU for biomolecules. Kathrine Nygaard Borg et al. [38] developed an Au-aptamer structure with a sensitivity of 1.5 × 10⁴ μm/RIU, achieving a detection limit of 1 aM for bovine serum albumin and 1 fM for TNF-α. These studies primarily focus on structural optimization to enhance sensing performance, while another promising approach involves refining optical configurations to further improve sensor capabilities.

Here, we introduce a beam displacement amplification technology (BDAT) in combination with SPR to amplify GH shifts and strengthen detection signals, thereby improving the sensitivity of the GH shift sensing system (Figure 1c). BDAT significantly amplifies the magnitude of the GH shift, achieving a 12-fold increase in our experiments. This application of BDAT improves the sensitivity of the sensing system by at least one order of magnitude. Compared to the method proposed by Li et al. [39], BDAT addresses challenges such as severe deformation of the beam intensity distribution (beam splitting) and the substantial reduction in GH shift signals typically observed in SPR-based systems. Furthermore, BDAT offers advantages such as ease of operation, presenting a promising new avenue for applying SPR sensing technology in high-precision detection fields.

## 2. Method and Experiment

**Figure 2:**
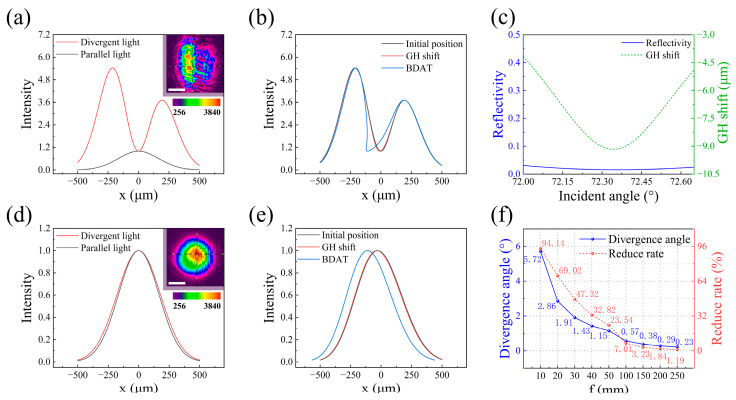
(**a**) Simulated intensity distributions for incident light focused by an objective lens versus parallel incident light. The inset shows the corresponding spot photograph. Scale bar for inset: 400 μm. (**b**) Simulation of the initial position of the light intensity distribution when the objective lens converges incident light, the light intensity distribution after GH shift, and the light intensity distribution after amplification by BDAT. (**c**) Magnified curves of reflectance and GH shift from Figure 1b. (**d**) Simulated intensity distributions for incident light focused by a convex lens *f* = 100 mm versus parallel incident light. The inset shows the corresponding spot photograph. Scale bar for inset: 400 μm. (**e**) Simulation of the initial light intensity distribution from the convex lens as it converges incident light, the light intensity distribution after GH shift, and the distribution after amplification by BDAT. (**f**) Divergence angles and GH shift reduction ratios for different focal lengths of O1.

In the beam displacement amplification method proposed by Li et al. [39], an objective lens is first used to focus the beam, and the reflected light is then magnified through an objective lens system to amplify the beam displacement. However, using the objective lens to focus the incident light causes the incident angle to no longer be fixed at the SPR angle, but instead spans a broader angular range (approximately 5.72°). Since the resonance absorption characteristics dependent on the incident angle of SPR (as shown by the blue line in Figure 1b), the reflectivity at this range does not change uniformly. The central reflectivity decreases more significantly, while the reflectivity at the sides decreases less, leading to a severe distortion of the intensity distribution of the spot, which no longer follows a Gaussian distribution, as shown in Figure 2a. Moreover, GH shift also depends on the incident angle. The maximum GH shift occurs at the angle corresponding to the strongest resonance absorption, and the magnitude of the GH shift rapidly decreases with changing angles. The point of maximum GH shift corresponds to the minimum intensity point in the spot’s intensity distribution, and the GH shift is confined to the weaker intensity regions (Figure 2b). As a result, the GH shift in this scenario is significantly reduced (approximately 94.14%).

To address these issues, the BDAT proposed in this study replaces the objective lens in the optical path with a convex lens (O1). Compared to the objective lens, the convex lens leads to a smaller divergence angle when focusing the beam. In this work, we used a convex lens with a focal length of 100 mm resulting in a divergence angle of only 0.57°. According to the SPR reflectivity curve, near the SPR angle, the reflectivity changes slowly (Figure 2c). Therefore, for incident light with a smaller divergence angle, the change in the reflectivity decay rate of the reflected spot is minimal, and the intensity distribution of the spot is less affected, remaining close to a Gaussian distribution (Figure 2d). Although GH shift changes rapidly near the SPR angle, the maximum GH shift occurs at the center of the spot’s intensity distribution, where the intensity is the highest, and the smaller GH shift occurs at the lower intensity regions at the sides of the spot. Therefore, the impact on GH shift size is minimal, with only a 7.01% reduction. BDAT, on the other hand, significantly amplifies the beam displacement. Figure 2e shows the position change of the spot before and after the GH shift, and after applying BDAT.

When using BDAT, the same spot is made to emit light at different positions and times, allowing for the localization of two distinct positions. The distance between these two positions corresponds to the beam displacement. In GH shift sensing measurements, the magnitude of GH shift varies with different sample solutions, resulting in a shift in the beam position. In Figure 1c, we represent the reflected light from different sample solutions as spots of different colors. The reflected light passes through a convex lens O2, which focuses it on the position-sensitive detector (PSD, PDP90A, Thorlabs, Newton, NJ, USA). The initial distance (L_1_) between the spots is amplified to a new distance (L_2_). By projecting the green and red spots onto the same PSD and obtaining their respective positions (Pr and Pg), the distance L_2_ between the spots can be measured. The magnification factor (*F*) is related to the object distance (*u*) and image distance (*v*): *F* = *v*/*u* The relationship between *u* and *v* follows the convex lens imaging formula: $\frac{1}{u}+\frac{1}{v}=\frac{1}{f}$, where *f* is the focal length of the convex lens (O2).

When selecting the incident convex lens (O1), two factors need to be considered: the divergence angle and the GH shift reduction ratio. As the focal length of O1 decreases, the divergence angle of the incident light increases. Simultaneously, due to the incident angle dependence of reflectivity and GH shift, the GH shift decreases compared to parallel light incidence, and the reduction ratio increases as the focal length of O1 decreases. In our experiment, we aim to achieve the largest possible divergence angle of the incident light to ensure that the reflected light passes through the optical center of the convex lens O2, thereby satisfying the BDAT conditions. Additionally, we need to ensure that the spot’s intensity distribution does not undergo severe distortion, keeping the GH shift reduction ratio within an acceptable range. The divergence angle and GH shift reduction ratio for various focal lengths of O1 are shown in Figure 2f, and the corresponding spot intensity distributions are illustrated in Figure S1. After considering these factors, we selected a convex lens O1 with a focal length of *f*_1_ = 100 mm.

## 3. Results and Discussion

### 3.1. Evaluation of Amplification Effect and Stability of BDAT

**Figure 3:**
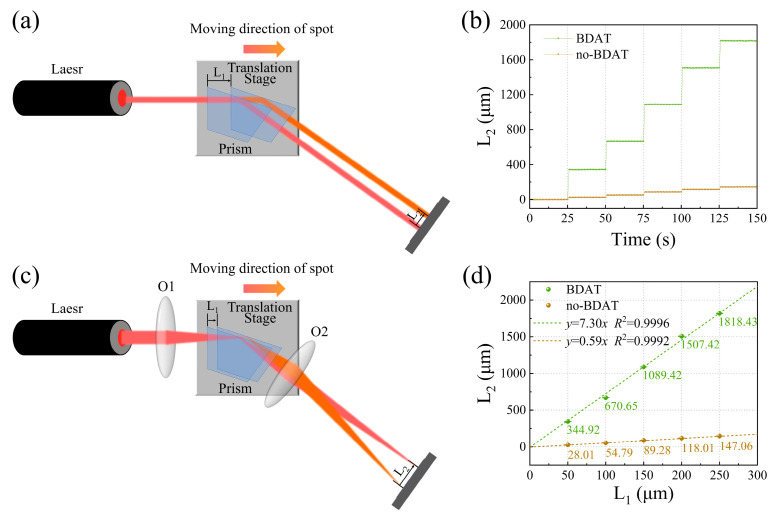
(**a**,**b**) Schematic of the experimental setup for evaluating the amplification effect and stability of BDAT. (**c**) L_2_ signal with and without BDAT. (**d**) Relationship between L_2_ and L_1_, along with linear fitting results.

To evaluate the amplification effect and stability of BDAT, we carried out a series of tests, which are shown in Figure 3a,b. The incident light (632.8 nm) was aligned parallel to the movement direction of the translation stage and perpendicular to the slanted surface of the trapezoidal prism. Therefore, the movement distance L_1_ of the translation stage corresponds to the displacement distance of the spot on the reflective surface of the prism. During the experiment, the translation stage was moved by 50 μm per 25 seconds. As the translation stage moved, light spots were sequentially projected and detected by the PSD, which determined the positions of the spots and obtained the spot displacement L_2_. Convex lenses O1 and O2 were added before and after the prism, respectively, and the experiment was repeated. The focal length of O2 was *f*_2_ = 100 mm, with an object distance *u* = 108 mm and an image distance *v* = 1300 mm. The theoretical magnification factor *F* was 12. The experimental results are shown in Figure 3c,d. Figure 3c presents the variation of L_2_ with time, while Figure 3d shows the relationship between L_2_ and L_1_. The dashed lines represent linear fits. Without BDAT, the slope *k*_1_ = 0.59, consistent with the geometric relationship between L_1_ and L_2_. With BDAT, the slope *k*_2_ = 7.30, yielding an experimental amplification factor *F* = *k*_2_/*k*_1_ = 12.37, which agrees well with the theoretical value. These results demonstrate the significant amplification effect and stability of BDAT. It should be noted that due to the action of O2, the sign of L_2_ before and after using BDAT is opposite. For clarity, the absolute values of L_2_ were used in the presentation.

### 3.2. Application of BDAT in GH Shift Sensing

**Figure 4:**
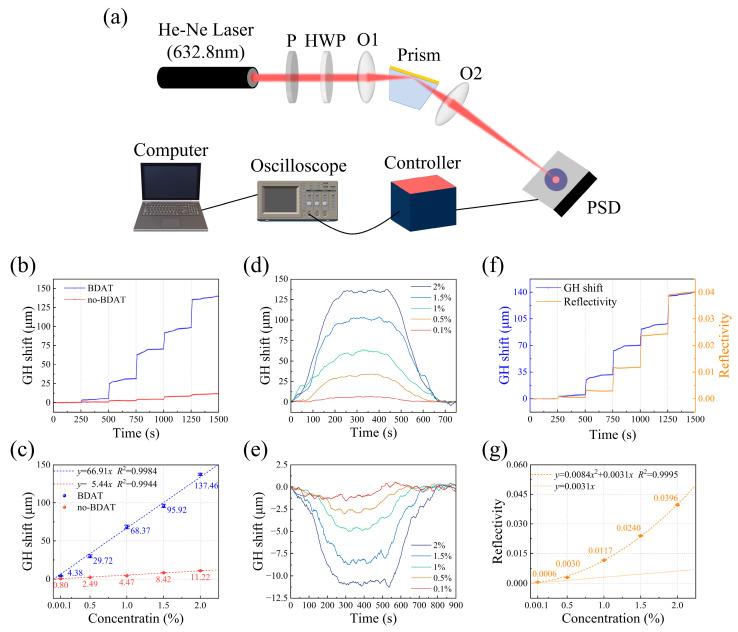
(**a**) Experimental schematic for GH shift sensing. P, polarizer; HWP, half-wave plate; O1, O2, convex lenses with *f* = 100 mm. (**b**) GH shift signals for NaCl solutions of different concentrations with and without BDAT. (**c**) Relationship between GH shift magnitude and NaCl concentration, along with linear fitting results. (**d**,**e**) GH shift signals for NaCl solutions of different concentrations were measured individually with and without BDAT. (**f**) Comparison of reflectivity change signals and GH shift change signals with BDAT for NaCl solutions of different concentrations. (**g**) Relationship between reflectivity change and NaCl concentration, along with polynomial fitting results.

Furthermore, we designed a series of experiments to demonstrate the application of BDAT in GH shift sensing. The experimental setup is illustrated in Figure 4a. The beam from a He-Ne laser was polarized into *p*-polarized light (polarization direction parallel to the incident plane) using a polarizer and a half-wave plate. The convex lens O1 focused the incident light onto the gold film-medium interface. The prism was mounted on a translation stage and an electronically controlled rotation stage, with the incident angle fixed at 72.19°, slightly smaller than the SPR angle. A trapezoidal prism with a base angle of 72° was used to ensure near-normal incidence and minimize adverse effects of refraction on the incident light quality. The sensing substrate was integrated with a microfluidic chamber, enabling convenient sample delivery using a syringe pump. The reflected beam was refocused by convex lens O2 onto the surface of a PSD. The real-time position of the reflected light was collected by the PSD, and the transverse position signal was recorded using a digital fluorescence oscilloscope. During sensing, when sample liquids were injected into the microfluidic chamber, the corresponding GH shift was measured in real-time.

We sequentially injected NaCl solutions with concentrations of 0.1%, 0.5%, 1%, 1.5%, and 2% into the microfluidic chamber at intervals of 250 seconds, monitoring the GH shift. Figure 4b shows the GH shift signals collected under different NaCl concentrations with (blue line) and without (red line) BDAT. Figure 4c shows the relationship between GH shift and NaCl concentration for both cases. The data exhibit an approximately linear relationship, and linear fitting was performed. Without BDAT, the slope *k*_1_ = 5.44, indicating that a 1% NaCl solution (0.00185 refractive index units, RIU) corresponds to a signal change of 5.44 μm, yielding a sensitivity *S*_1_ = 2.94 × 10^3^ μm/RIU. With BDAT, the slope *k*_2_ = 66.91, resulting in a sensitivity *S*_2_ = 3.62 × 10^4^ μm/RIU, which represents an order-of-magnitude improvement. The amplification factor *F* = 12.30 is consistent with the theoretical value, confirming the effectiveness of BDAT in enhancing GH shift-sensing sensitivity.

According to the measurement results for the NaCl solution with zero concentration, the standard deviation without BDAT is σ_1_ = 0.11809 μm, and the corresponding resolution is R_1_ = σ_1_/S_1_ = 4.02 × 10⁻⁵ RIU. When BDAT is applied, the standard deviation is σ_2_ = 0.09364 μm, and the resolution improves to R_2_ = σ_2_/S_2_ = 2.59 × 10⁻⁶ RIU. Therefore, the application of BDAT not only avoids increasing the standard deviation but also significantly enhances the resolution.

We also conducted measurements for NaCl solutions of varying concentrations. During the experiment, water was first injected into the microfluidic chamber, followed by the NaCl solution, and then water again. The experimental results before and after using BDAT are shown in Figure 4d and Figure 4e, respectively. As the NaCl solution in the injection tube was gradually diluted by water, the GH shift signal induced by the NaCl solution changed slowly, reaching saturation at approximately 250 s. This saturated state lasted for about 200 s before the GH shift changed again due to water injection, ultimately returning to its initial state. The relationship between the GH shift magnitude and NaCl concentration under both conditions is summarized in Figure S2a. The results are highly consistent with those obtained during “unified measurements”, indicating that BDAT exhibits excellent stability.

Additionally, we employed the traditional SPR intensity mode for measurements, using a fixed-angle configuration. The experimental procedure was identical to that for “unified measurements” of GH shift signals, except that the PSD was replaced with an Optical Power Meter to collect intensity signals and calculate reflectivity. The reflectivity variation results obtained were compared with the GH shift variations measured using BDAT, as shown in Figure 4f. The relationship between reflectivity changes and NaCl concentration is presented in Figure 4g. The two exhibit a nonlinear relationship, and a polynomial fit was performed, as indicated by the dashed line. The solid line in the figure represents the tangent to the dashed line at *x* = 0, used to evaluate the sensor’s sensitivity in the ultra-low concentration range. The slope *k* = 0.00311, resulting in a sensitivity *S* = 1.68 a.u./RIU.

To evaluate the sensing performance of the two modes, the minimum detection limit *D* can be calculated using the formula *D* = 3*N*/*S*, where *N* is the noise level and *S* is the sensitivity [40]. To assess sensor performance in ultra-low concentration ranges, *N* is uniformly taken as the noise level at zero concentration. For GH shift sensing using BDAT, *D*_1_ = 3.10 × 10^−5^ RIU. For the traditional intensity-based SPR sensing mode, *D*_2_ = 5.3 × 10^−5^ RIU. This demonstrates that GH shift sensing has a lower theoretical detection limit and offers superior performance in ultra-low concentration sensing.

### 3.3. Comparative Analysis of GH Shift and Reflectivity for Low-Concentration Sensing

**Figure 5:**
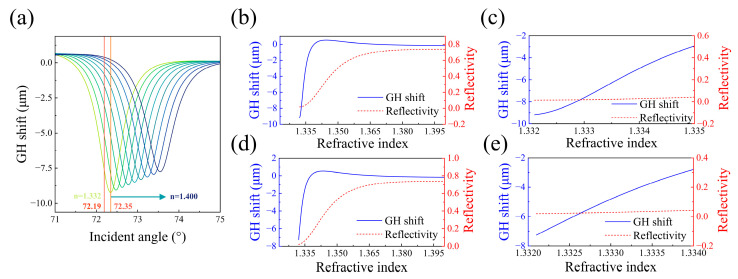
(**a**) GH shift curves for different refractive indices. (**b**) GH shift and reflectivity change curves for varying refractive indices at a fixed incident angle of the SPR angle (72.35°). (**c**) Magnified view of the curves in (**b**). (**d**) GH shift and reflectivity change curves for varying refractive indices at a fixed incident angle slightly smaller than the SPR angle (72.19°). (**e**) Magnified view of the curves in (**d**).

As shown in Figure 5a, the GH shift resonance peak shifts toward larger incident angles as the refractive index of the medium increases. If the incident angle is set at the SPR angle (72.35°), the maximum GH shift can be achieved. However, as depicted in Figure 5b, the curve of GH shift versus refractive index exhibits nonlinear growth in the low-concentration region, and the GH shift increases more slowly in the ultra-low concentration range (Figure 5c). If the incident angle is fixed slightly below the SPR angle, such as at 72.19°, the GH shift magnitude and linear range decrease slightly, but the curve remains linear in the low-concentration region, and the GH shift increases more rapidly, as shown in Figure 5d,e. Compared to the GH shift curve, the slope of the reflectivity curve is much smaller and exhibits a more pronounced nonlinear relationship in the low-concentration region. Reflectivity changes are significantly slower than GH shifts. Thus, in theory, GH shift sensing is more suitable for measuring low-concentration solutions.

## 4. Conclusions

In this work, we combined BDAT with SPR to enhance GH shift sensing, significantly improving the sensitivity of the sensing system by amplifying the beam shift. By replacing the objective lens with convex lenses, BDAT addresses the issues caused by the method of Li et al. [39], which when applied to SPR systems, resulted in severe distortion of beam intensity distribution and substantial reduction in GH shift signals. Refractive index sensing experiments with NaCl solutions of varying concentrations demonstrate that BDAT achieves significant enhancement of GH shifts with a highly stable beam displacement amplification factor *F*. The sensitivity achieved is 3.62 × 10^4^ μm/RIU, with a maximum detectable lateral displacement of 137.46 μm and a theoretical minimum detection limit of 3.10 × 10^−5^ RIU. Its sensitivity level has reached the same order of magnitude as previous publications. Furthermore, both theoretical and experimental results confirm that, compared to traditional intensity-based SPR sensing, GH shift sensing offers superior performance in measuring low-concentration solutions. In summary, the BDAT proposed in this study has been demonstrated to amplify GH shifts by at least 12-fold, significantly enhancing sensitivity. Additionally, the advantages of GH shift sensing in low-concentration solution measurements have been thoroughly validated. Our method provides a practical and reliable performance enhancement strategy for SPR-based sensors. In future research, further performance improvements can be achieved by optimizing the thin-film structure and related parameters. We believe that the stable BDAT holds great potential for improving the sensitivity of GH shift-based biosensors. The GH shift sensing system presented in this work has broad application prospects in fields such as biochemical analysis and environmental monitoring.

## Data Availability

All of the data that support the findings of this study are reported in the main text and Supplementary Materials. Source data are available from the corresponding author upon reasonable request. Correspondence and requests for materials should be addressed to Z. B. Liu.

## Supplementary Materials

The following supporting information can be downloaded at: https://www.mdpi.com/article/10.3390/s25051329/s1. Table S1. Thickness and RI of each material. Figure S1. Light intensity distribution of light spots corresponding to O1 with different focal lengths (a) f = 250 mm (b) f = 200 mm (c) f = 150 mm (d) f = 100 mm (e) f = 50 mm (f) f = 40 mm (g) f = 30 mm (h) f = 20 mm (i) f = 10 mm. Figure S2. Relationship between GH shift magnitude and NaCl concentration during individual (a) and unified (b) measurements, along with linear fitting results. References [41,42,43,44,45,46,47] are cited in the supplementary materials.
