# Supplementary material for: High-Sensitivity Goos-Hänchen Shift Sensing via Surface Plasmon Resonance and Beam Displacement Amplification
Source: Sensors (Basel). 2025 Feb 21;25(5):1329. doi: 10.3390/s25051329 (PMC11902807; doi:10.3390/s25051329)
Supplement: Supplementary file 1 [file sensors-25-01329-s001.zip › sensors-3453784-supplementary.pdf]

Supporting Information for

High-Sensitivity Goos-Hänchen Shift Sensing via Surface Plasmon Resonance and Beam Displacement Amplification

Qian Li [1,2,†], Enze Xu [1,2,†], Xiaoliang Zhang [3], Jianguo Tian [1,2] and Zhibo Liu [1,2,4,*]

1 The Key Laboratory of Weak Light Nonlinear Photonics, Ministry of Education, School of Physics and Teda Applied Physics Institute, Nankai University, Tianjin 300071, China; 2120230287@mail.nankai.edu.cn (Q.L.); stevenxez@gmail.com (E.X.); jjtian@nankai.edu.cn (J.T.)
2 State Key Laboratory of Photovoltaic Materials and Cells, Nankai University, Tianjin 300071, China
3 Institute of Biomedical Precision Testing and Instrumentation, College of Artificial Intelligence, Taiyuan University of Technology, Jinzhong 030600, China; zhangxiaoliang@tyut.edu.cn
4 The Collaborative Innovation Center of Extreme Optics, Shanxi University, Taiyuan 030006, China
* Correspondence: liuzb@nankai.edu.cn
† These authors contributed equally to this work.

S1. Material Parameters of the Plasmonic Sensing Substrate

Table S1. Thickness and RI of each material

Material	Thickness(nm)	RI(λ=632.8nm)
BK7 glass	/	1.515
Cr	2	3.136 + 3.312i
Gold film	50	0.183 + 3.431i
Sensing medium	/	1.332

S2. Theoretical Calculation for Reflectivity, Phase, and GH Shift

In a Kretschmann configuration, the reflection coefficient of two adjacent layers takes the form of

$$\mathrm{r}_{i,i+1} = \frac{Z_i - Z_{i+1}}{Z_i + Z_{i+1}}, \tag{S1}$$

where $\mathrm{Z}_{\mathrm{ip}} = \dfrac{\varepsilon_i}{k_i}$ for p polarization and $\mathrm{Z}_{\mathrm{is}} = k_i$ for s polarization。 ε_i represents the complex dielectric constants of the i-th layer and $k_i = k_0\sqrt{\varepsilon_i - \varepsilon_1 \sin^2\theta_c}$, in which k_0 is the wave vector of the optical wave in free space and θ_c is the incident angle.

For a sensing substrate with m layers, we have

$$r_{m-2,m} = \frac{r_{m-2,m-1} + r_{m-1,m}\exp(2ik_{m-1}d_{m-1})}{1 + r_{m-2,m-1}r_{m-1,m}\exp(2ik_{m-1}d_{m-1})}. \tag{S2}$$

Then, we subsequently calculate $r_{m-3,m}, r_{m-4,m} \cdots$ until we obtain $r_{1,m}$, which is the reflectivity of the substrate based on this structure.

From Fresnel's equations, the complex reflection coefficients can be expressed as $r_{p(s)} = |r_{p(s)}|\exp(i\phi_{p(s)})$ for p and s polarizations in which $\phi_{p(s)}$ represents the phase of both polarizations. According to the stationary phase approach[1], the GH shift represents the higher order mode of the phase signal [2-4] and can be determined through the following equation[5]:

$$\Delta_{GH} = -\frac{1}{k_0}\frac{\partial \phi}{\partial \theta}. \tag{S3}$$

If we determine the reflection coefficients through the standard characteristic matrix approach[6,7], the GH shift can also be expressed as:

$$\Delta_{GH} = -\frac{\lambda}{2\pi\left|r_{p(s)}\right|^2}\left[\mathrm{Re}(r_{p(s)})\frac{d\,\mathrm{Im}(r_{p(s)})}{d\theta} - \mathrm{Im}(r_{p(s)})\frac{d\,\mathrm{Re}(r_{p(s)})}{d\theta}\right], \tag{S4}$$

where Re is the real part and Im is the imaginary part. Based on the above equations we can obtain the GH shift.

S3. Simulation of Light Spot Intensity Distribution

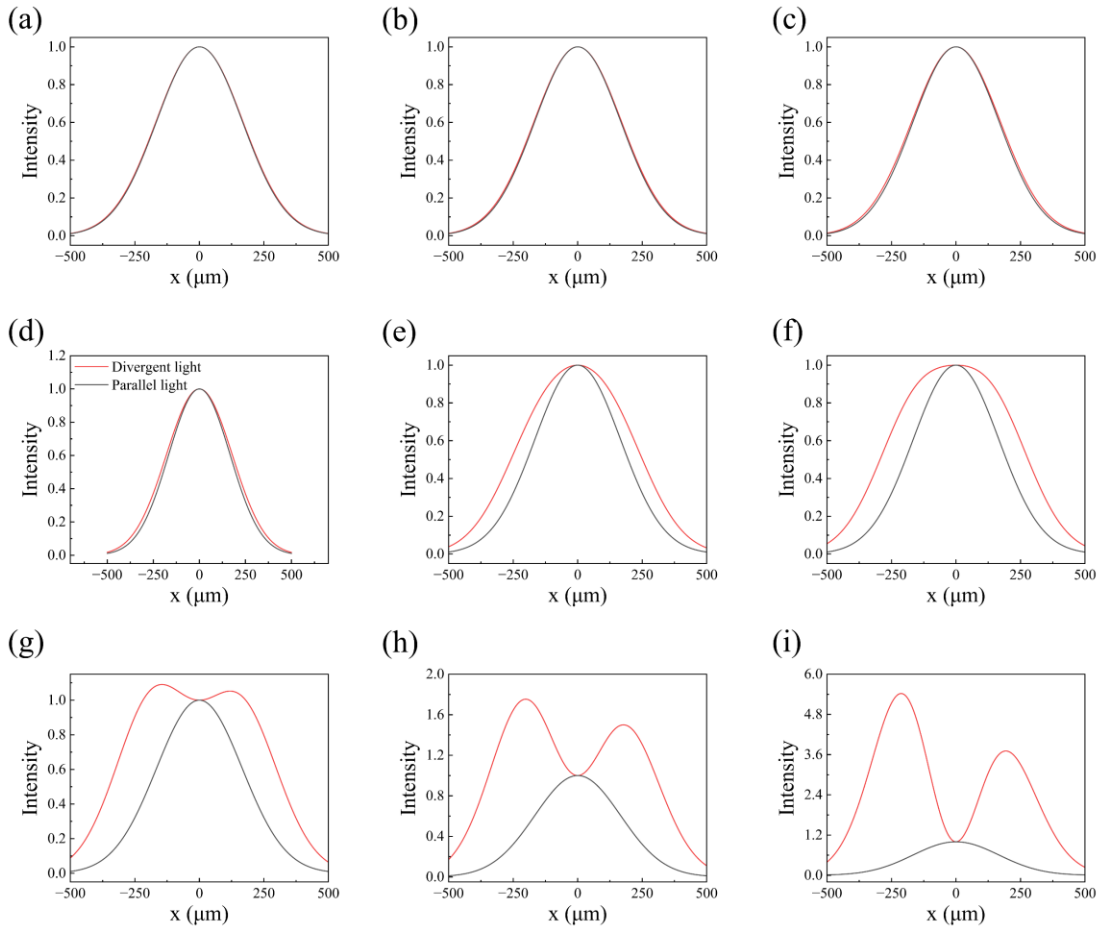

Figure S1. Light intensity distribution of light spots corresponding to O1 with different focal lengths (a) f = 250 mm (b) f = 200 mm (c) f = 150 mm (d) f = 100 mm (e) f = 50 mm (f) f = 40 mm (g) f = 30 mm (h) f = 20 mm (i) f = 10 mm

S4. Detection of NaCl Solution

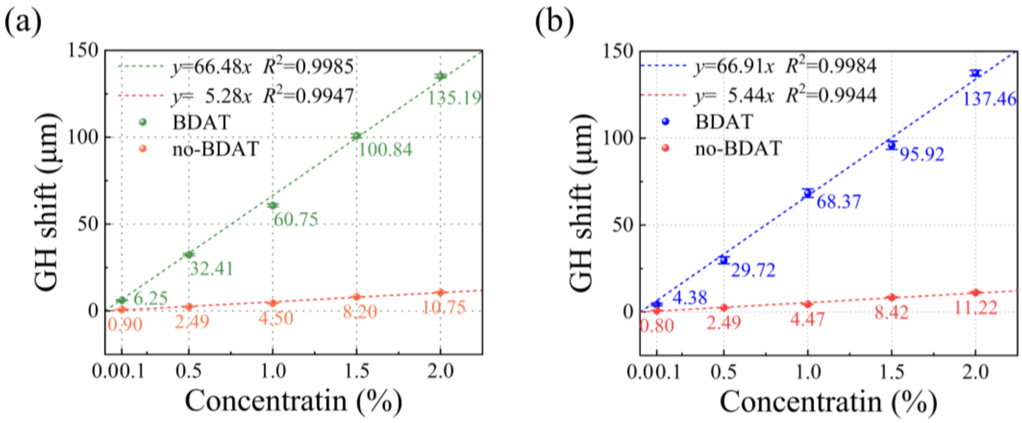

Figure S2. Relationship between GH shift magnitude and NaCl concentration during individual (a) and unified (b) measurements, along with linear fitting results.

References

1. Li, C.F. Negative lateral shift of a light beam transmitted through a dielectric slab and interaction of boundary effects. Phys. Rev. Lett. 2003, 91, doi:10.1103/PhysRevLett.91.133903.
2. Feng, F.; Si, G.; Min, C.; Yuan, X.; Somekh, M. On-chip plasmonic spin-Hall nanograting for simultaneously detecting phase and polarization singularities. Light Sci. Appl. 2020, 9, 95-95, doi:10.1038/s41377-020-0330-z.
3. Malassis, L.; Masse, P.; Treguer-Delapierre, M.; Mornet, S.; Weisbecker, P.; Barois, P.; Simovski, C.R.; Kravets, V.G.; Grigorenko, A.N. Topological Darkness in Self-Assembled Plasmonic Metamaterials. Adv. Mater. 2014, 26, 324-330, doi:10.1002/adma.201303426.
4. Yesilkoy, F.; Terborg, R.A.; Pello, J.; Belushkin, A.A.; Jahani, Y.; Pruneri, V.; Altug, H. Phase-sensitive plasmonic biosensor using a portable and large field-of-view interferometric microarray imager. Light Sci. Appl. 2018, 7, doi:10.1038/lsa.2017.152.
5. Wu, W.; Zhang, W.; Chen, S.; Ling, X.; Shu, W.; Lu, H.; Wen, S.; Yin, X. Transitional Goos-Hanchen effect due to the topological phase transitions. Opt. Express 2018, 26, 23705-23713, doi:10.1364/oe.26.023705.
6. Luo, C.; Guo, J.; Wang, Q.; Xiang, Y.; Wen, S. Electrically controlled Goos-Hanchen shift of a light beam reflected from the metal-insulator-semiconductor structure. Opt. Express 2013, 21, 10430-10439, doi:10.1364/oe.21.010430.
7. Lee, S.; Li, L. Rapid super-resolution imaging of sub-surface nanostructures beyond diffraction limit by high refractive index microsphere optical nanoscopy. Opt. Commun. 2015, 334, 253-257, doi:10.1016/j.optcom.2014.08.048.
